# Retroperitoneal Hematoma as a Complication of Endovascular Embolization of Tumor Epistaxis: A Case Report

**DOI:** 10.7759/cureus.20759

**Published:** 2021-12-27

**Authors:** Mia Benavidez, Nicholas A Rossi, Jordan W Rawl, Mohamad R Chaaban

**Affiliations:** 1 Medicine, University of Texas Medical Branch, Galveston, USA; 2 Otolaryngology-Head and Neck Surgery, University of Texas Medical Branch, Galveston, USA; 3 Otolaryngology, Lexington ENT and Allergy, Lexington Medical Center, West Columbia, USA; 4 Otolaryngology, Head and Neck Institute, Cleveland Clinic, Cleveland, USA

**Keywords:** retroperitoneal hematoma, perinephric hematoma, tumor epistaxis, interventional radiology guided embolization, endovascular embolization

## Abstract

Retroperitoneal hematomas are a rare and fatal complication of endovascular embolization. We report a case of an 89-year-old woman who was referred to interventional radiology for percutaneous embolization for intractable epistaxis as a result of a left nasal cavity mucosal melanoma. After successful embolization of the left sphenopalatine artery, the patient became hypotensive and was transferred to the intensive care unit. Post-operative CT abdomen and pelvis angiogram showed a large right perinephric hematoma, which is an extremely uncommon complication of endovascular embolization for epistaxis. Practitioners should be aware of this life-threatening complication in weighing the risks and benefits of embolization versus direct surgical ligation, and they should identify and intervene promptly if a retroperitoneal hematoma should occur.

## Introduction

Retroperitoneal hematomas remain rare and life-threatening with mortality rates reported between 18-60% [1]. Cases of retroperitoneal hematomas in patients following interventional procedures were reported to occur in less than 3% and in 1.85% of those undergoing endovascular embolization for epistaxis [2,3].

Endovascular embolization is a well-known procedure used for the past 30 years to treat intractable epistaxis with a high success rate of around 88% and is an especially suitable alternative for treatment of epistaxis when surgery is contraindicated [4]. PubMed literature search revealed one prior article citing retroperitoneal hematoma as a complication of endovascular embolization for epistaxis [2]. Thus, due to this lack of literature reporting, many practitioners may not be adequately prepared to recognize retroperitoneal hematoma as a potential complication of that procedure. Here, we present an unusual case of a retroperitoneal hematoma emerging after a successful embolization of the left sphenopalatine artery to treat tumor epistaxis.

This article was previously presented as a poster at the 2018 American Academy of Otolaryngology-Head and Neck Surgery (AAO-HNSF) Annual Meeting and OTO Experience in Atlanta, Georgia, on October 7-10, 2018.

## Case presentation

**Figure 1:**
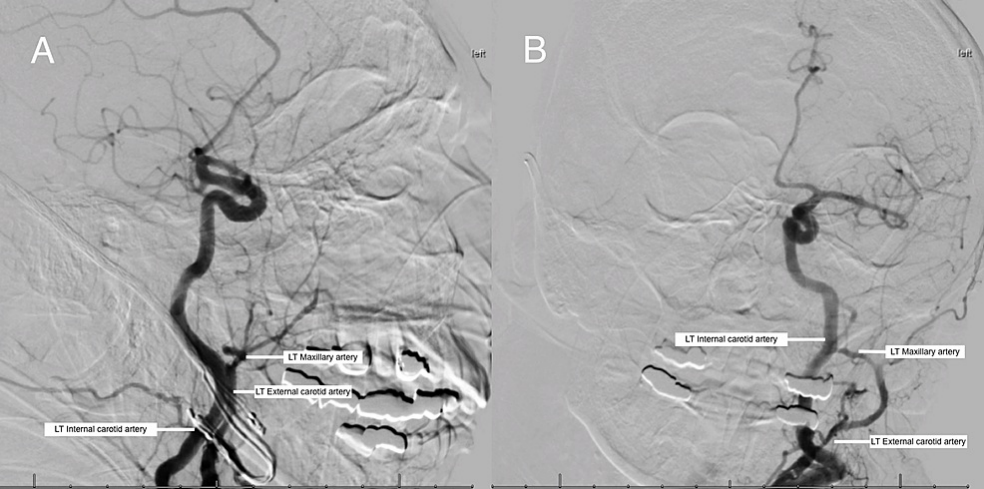
Embolization of left sphenopalatine artery (A and B). The above fluoroscopy images demonstrate successful embolization of multiple hypertrophied septal branches of the left sphenopalatine artery (branch of the maxillary artery). No residual blush is seen within the left nasal cavity, confirming complete embolization of bleeding vessels. LT: left; RT: right

An 89-year-old woman with a past medical history of advanced Alzheimer’s disease, hypertension, and T3N0M0 recurrent left nasal cavity mucosal melanoma presented with unrelenting epistaxis despite conservative measures, causing severe disturbance in quality of life. Direct surgical control of the bleeding was considered. However, due to the patient’s significant medical comorbidities, the decision was made to undergo endovascular embolization of the left sphenopalatine artery on May 26, 2017 (Figures 1A, 1B). The procedure was uncomplicated, and the artery was successfully embolized with 300-500 um Embosphere Microspheres (Jordan, UT: Merit Medical Systems, Inc.) and Gelfoam (New York City, NY: Pfizer Inc.) (accessed via the right common femoral artery with an 8 French sheath {Bloomington, IN: Cook Group Incorporated} and closed with a 6 French Perclose Proglide {Chicago, IL: Abbot Laboratories}), which led to cessation of the bleeding. Ten minutes later, in the post-anesthesia care unit, she became lethargic, obtunded, and subsequently non-responsive. Her radial pulse was weak and thready.

The patient’s vital signs showed severe hypotension with blood pressures in 60s/40s. Chest x-ray showed mild bilateral atelectatic changes, but this and abdominal x-ray were otherwise normal. Arterial blood gas showed pH of 7.28, partial pressure of carbon dioxide (pCO_2_) of 34 mmHg, partial pressure of oxygen (pO_2_) of 105 mmHg, and bicarbonate level (HCO3) of 16 mEq/L. Urgent hemogram showed a hemoglobin of 7.0 g/dL, a 40% decrease from her pre-operative value of 11.9 g/dL. Her electrolytes and urinalysis were within normal limits. Vital signs were unstable, precluding opportunity to obtain computed tomography (CT).

Massive transfusion protocol was initiated, and multiple units of packed red blood cells were administered. The patient was brought to the operating room by vascular surgery team for an emergent pelvic angiogram, but no extravasation of contrast was noted. Intra-operatively, the patient experienced frequent episodes of hypotension, with the lowest blood pressure recorded as 57/39. Intra-operative hemogram showed the patient’s hemoglobin to be 5.1 g/dL, the lowest it would reach during admission. She responded to intra-operative administration of two vasopressors, ephedrine and phenylephrine. The vascular surgery team performed a thorough angiogram, but no active bleeding could be identified. Her vital signs eventually stabilized intra-operatively with aggressive administration of vasopressors and packed red blood cells, and she was transported to the surgical intensive care unit (Table 1).

**Table 1:** Vital signs timeline. bpm: beats per minute

Vital Signs	Blood Pressure	Heart Rate
Baseline	129-165/68-87	64 bpm
Initial presentation	209/71	59 bpm
Intra-operatively (first procedure)	81/62	58 bpm
Immediately post-operatively	85/61	68 bpm
Ten minutes post-operatively	79/52	48 bpm
Intra-operatively (second procedure)	124/87	55 bpm
Post-operatively	151/110	59 bpm
Discharge	182/74	80 bpm

**Figure 2:**
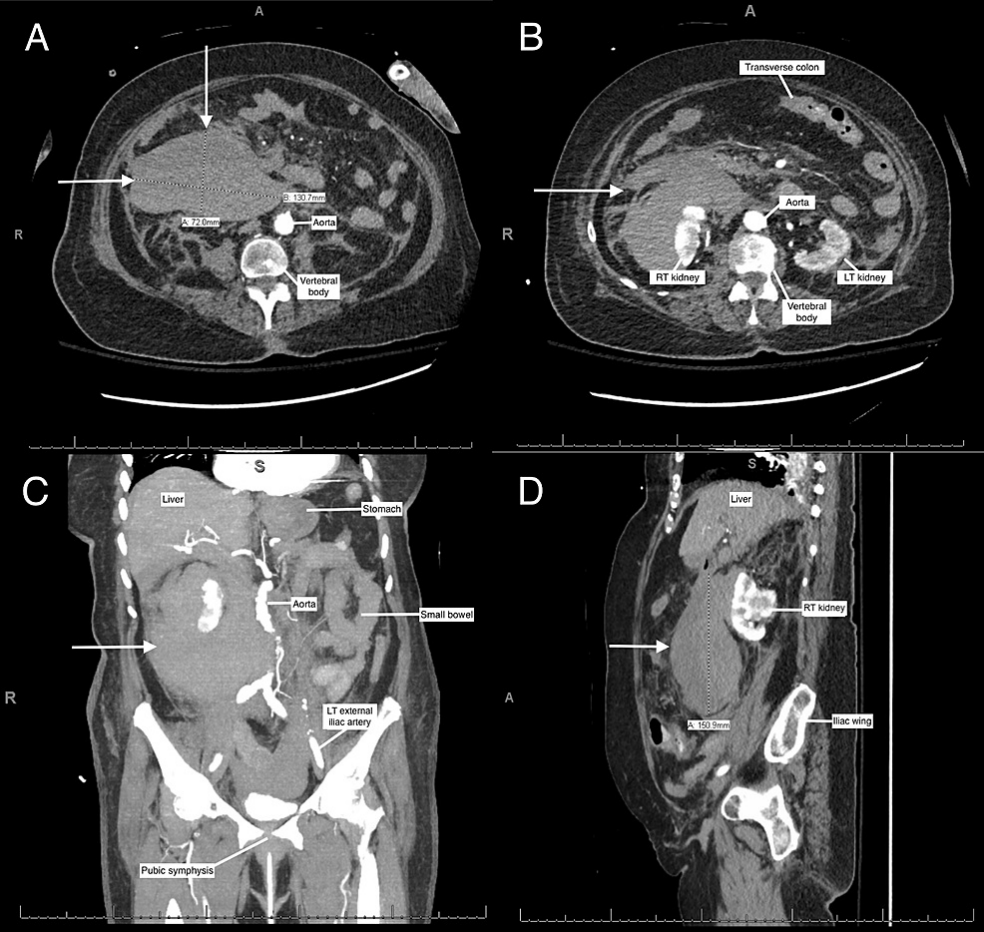
CT abdomen and pelvis of right subcapsular perinephric hematoma (A-D). CT abdomen and pelvis angiogram demonstrating a large right subcapsular perinephric hematoma measuring 7.2x13.1x15.1 cm in (A and B) axial plane, (C) coronal plane, and (D) sagittal plane (white arrows: perinephric hematoma). CT: computed tomography; LT: left; RT: right

When her vital signs had stabilized appropriately, post-operative CT abdomen and pelvis angiogram revealed a large right retroperitoneal and perinephric hematoma (Figures 2A-2D). She was treated conservatively with close monitoring in the surgical intensive care unit. The patient was slowly weaned off vasopressors and discharged to a skilled nursing facility one week post-operatively. She was seen in clinic six weeks following discharge and found to be recovered well surgically with no signs of further epistaxis or concerns for hemodynamic instability. However, her mental status had severely declined since discharge, which had been attributed to a rapid progression in her Alzheimer’s disease. Her family elected to pursue palliative care, and the patient passed away shortly thereafter.

## Discussion

Retroperitoneal hematomas, defined as hemorrhage in the retroperitoneal space, are usually associated with blunt injuries or ruptured blood vessels within the abdomen and pelvis. In the setting of femoral artery catheterizations, the incidence of retroperitoneal hematomas reported as a complication of the procedure is around 0.5% [5]. Higher femoral artery puncture sites (i.e., above the middle third of the femoral head on fluoroscopy) is a known risk factor for retroperitoneal hematomas [5-7]. A greater predisposition to uncontrolled bleeding is evident if the puncture site was above the inguinal ligament and into the external iliac artery. Additional risk factors include female gender, low body surface area, and anticoagulative medications [6,7]. In reference to this case, the patient was female, of average body surface area for an adult (1.701 m^2^), and was not taking anticoagulative medications. However, it is unclear where the specific puncture site of the procedure occurred.

Signs and symptoms of retroperitoneal hematomas from interventional procedures can include suprainguinal tenderness and fullness, severe back and lower quadrant abdominal pain, and femoral neuropathy [7]. Because the retroperitoneum can hold enough blood to mask external presentation, the recognition of this complication is often delayed until the patient becomes hypotensive [6]. Whenever a patient cannot verbalize their pain or when the diagnosis is not apparent from physical examination, definitive diagnosis is made with abdominal CT scan, such as in this case where the patient became hypotensive following the procedure, and pelvic angiogram failed to demonstrate extravasation of contrast [7]. When managing a retroperitoneal hematoma, early identification can improve patient outcomes since maintaining hemodynamic stability is critical in preventing fatal hemorrhage or shock [8]. For patients with altered consciousness following the procedure, post-operative evaluation may include a thorough physical examination, obtaining routine measurements of hematocrits, and closely tracking blood pressure [3]. Surgery is rarely used as therapy for a retroperitoneal hematoma and should only be reserved for those who are unresponsive to treatment. In this case, as in most cases, the patient’s symptoms were successfully managed by volume resuscitation and transfusion [7].

When considering treatment options for intractable epistaxis, surgical ligation and endovascular embolization of the bleeding artery are often weighed against one another. The main advantage of endovascular embolization is the potential for avoidance of general anesthesia or endotracheal intubation, occlusion of other vessels contributing to the epistaxis, diagnosis of other vascular abnormalities, and less surgical disruption to the nasal mucosa [9]. These advantages provide sufficient considerations for a patient, such as in this case, who is a poor surgical candidate because of age and other comorbidities. Minor complications occur in 8-13% of endovascular embolization of epistaxis, the most common being rebleeding [4]. Major complications of endovascular embolization arise in around 0-7% of patients and include cerebrovascular accident, central retinal artery occlusion, hypoxia, hypovolemia, angina with or without myocardial infarction, and retroperitoneal hemorrhage [2,4]. This case illustrates a major complication that should be further considered when surgery is contraindicated in the treatment of intractable epistaxis.

## Conclusions

Retroperitoneal hematomas are a rare complication that can result from interventional procedures and known risk factors include higher femoral artery puncture site, female gender, low body surface area, and anticoagulative medications. Practitioners should be prepared to recognize and promptly intervene in the case of retroperitoneal hematoma as a potential complication of endovascular embolization. Additionally, the risks and benefits of embolization versus direct surgical ligation must be heavily weighed on a case-by-case basis.
